# Necrology

**Published:** 1902-07

**Authors:** 


### NECROLOGY.

DR. CHARLES FAIVRE, of 2435 Fairmount Avenue, Philadelphia, Pa., an old and esteemed practitioner, died in June, aged sixty-five years. Dr. Faivre was born in France and received his training in that country. He spent five years in Africa under the French foreign veterinary service. He leaves a wife and five children.

